# Liver Enzymes Correlate With Metabolic Syndrome, Inflammation, and Endothelial Dysfunction in Prepubertal Children With Obesity

**DOI:** 10.3389/fped.2021.629346

**Published:** 2021-02-16

**Authors:** Rosario Valle-Martos, Miguel Valle, Rosario Martos, Ramón Cañete, Luis Jiménez-Reina, María Dolores Cañete

**Affiliations:** ^1^Maimonides Biomedical Research Institute of Córdoba, University of Cordoba, Córdoba, Spain; ^2^Valle de los Pedroches Hospital, Maimonides Biomedical Research Institute of Córdoba, Córdoba, Spain; ^3^Health Center of Pozoblanco, Maimonides Biomedical Research Institute of Córdoba, Córdoba, Spain; ^4^Faculty of Medicine, Maimonides Biomedical Research Institute of Córdoba, Biomedical Research Networking Center for Physiopathology of Obesity and Nutrition, University of Cordoba, Córdoba, Spain; ^5^Faculty of Medicine, Maimonides Biomedical Research Institute of Córdoba, University of Cordoba, Córdoba, Spain; ^6^Maimonides Biomedical Research Institute of Córdoba, Cordoba, Spain

**Keywords:** liver enzymes, prepuberal age, obesity, inflammation, metabolic syndrome, endothel dysfunction

## Abstract

**Background:** Metabolic syndrome (MetS) can start in children with obesity at very young ages. Non-alcoholic fatty liver disease (NAFLD) is considered to be the hepatic component of metabolic syndrome. If left untreated, the clinical course of NAFLD can be progressive and can become chronic if not detected at an early stage.

**Objective:** We aimed to quantify the differences in liver enzymes between prepubertal children with obesity and children with normal weight to determine any associations between them and parameters related to MetS, adipokines, or markers of endothelial dysfunction and inflammation.

**Methods:** This cross-sectional study included 54 prepuberal children with obesity (aged 6–9 years) and 54 children with normal weight, matched by age and sex. Liver enzymes, C-reactive protein (CRP), interleukin-6, soluble intercellular adhesion molecule-1 (sICAM-1), adipokines, and parameters related to metabolic syndrome (MetS) were all measured.

**Results:** Alanine aminotransferase (ALT) levels, serum butyryl cholinesterase (BChE), leptin, CRP, sICAM-1, triglycerides, blood pressure, and homeostasis model assessment for insulin resistance were significantly higher in children with obesity, while Apolipoprotein A-1, HDL-cholesterol, and adiponectin were significantly lower. In the children with obesity group, ALT and BChE levels correlated with anthropometric measurements, insulin resistance, and lipid parameters, leptin, interleukin-6, CRP, and sICAM-1 while BChE levels negatively correlated with adiponectin.

**Conclusions:** Compared to children with normal weight, prepubertal children with obesity had elevated values for liver enzymes, leptin, markers of insulin resistance, inflammation, and endothelial dysfunction, and variables associated with MetS. There was also a correlation between these disorders and liver enzyme levels.

## Introduction

Metabolic syndrome (MetS) can start in obese children at very young ages (1), and a not negligible proportion of prepubertal children were even classified as affected by metabolic syndrome in the IDEFICS study (2). The presentation of this syndrome in children is associated with a high risk for diabetes and atherosclerotic cardiovascular disease during adulthood (3).

Together with the disorders that define metabolic syndrome, a subclinical inflammation state indicative of alterations producing endothelial dysfunction, as well as changes in adipokine levels, have been documented in prepubertal children with obesity (4–6).

Non-alcoholic fatty liver disease (NAFLD) is associated with important components of MetS such as obesity (7, 8), some authors consider these to be hepatic manifestations of this syndrome (9) and its prevalence increases in line with that of MetS and its components, particularly obesity (10). NAFLD has now become the most frequent cause of chronic liver disease, both in children and adults (10, 11).

The pathogenesis of NAFLD is multifactorial, dietary factors, insulin resistance (IR), inflammation, adipocytokines, lipotoxicity, and a genetic predisposition, are all factors that may be involved (12).

Elevated Alanine aminotransferase (ALT) levels are associated with the incidence of MetS, diabetes mellitus, and cardiovascular disease and have been shown to be a predictive factor for non-alcoholic steatosis (13, 14). Elevated serum butyryl cholinesterase (BChE) levels have also been described in individuals with obesity and MetS (15) and there is an association between BChE activity, lipid metabolism, and MetS in adults (16).

Adipose tissue plays an important role in the pathogenesis of NAFLD by contributing to the low-grade inflammation strongly related to this disease (17). In a subsample of European children forming part of the IDEFICS study, the highest levels of leptin were associated with metabolic syndrome, regardless of body mass. The measurement of leptin could help to discriminate overweight/obese children with an increased risk of cardio-metabolic complications while still at an early age (18).

The available data indicate that obesity-associated metabolic disorders, including NAFLD, IR, and inflammation, as well as altered levels of adipokines, can onset at very early ages. Although limited, the data on the natural history of pediatric NAFLD show that a few children rapidly progress from NAFLD to clinical events, some of which are even more severe than those diagnosed in adults (19, 20). This emphasizes the potential importance of applying preventive interventions in patients while they are still young, and consequently, the need for diagnostic tools that are easy to use in daily clinical practice.

Limited information is available about the possible association between liver-function enzymes, obesity-related disorders, and NAFLD in prepubescent children. There is currently a lack of predictors of NAFLD among young children with obesity. The seriousness of these pathologies and the consequences of their clinical evolution without treatment serve to highlight the need for their early diagnosis and treatment. Therefore, we must try to develop diagnostic tools that can be applied in general clinical practice.

Thus, in this study we aimed to analyse the possible differences between children with obesity and prepubertal children with normal weight in liver enzyme values to determine any associations between them and parameters related to MetS, adipokines, and markers of endothelial dysfunction or inflammation.

## Materials and Methods

### Study Design

We carried out a cross-sectional study in prepubertal (Tanner stage 1) children with obesity of both sexes. To reduce selection bias, both groups, cases and controls were selected from the same population, and case control ratio observed was of 1:1, matched by age and sex.

Inclusion criteria were the same for the study group and control group with the exception for BMI: prepubertal (Tanner stage 1) children of both sexes and aged 6–9 years. The children were placed into one or the other group according to whether their BMI categorized them as with obesity (cases) or normoweight (control).

Exclusion criteria for both groups: children with diabetes (fasting glucose ≥ 7.0 mmol/L), impaired fasting glucose (fasting glucose ≥ 6.1 mmol/L or < 7.0 mmol/L), primary hyperlipidaemia, hypertension, aspartate aminotransferase >40 U/L, or secondary obesity were excluded from both the study. None of the participants were receiving any regular treatments with any medications. Children with CRP levels > 10 mg/L (which thus indicates the presence of clinically relevant inflammatory conditions), were excluded from both the children with obesity group and the control group. ALT levels must be interpreted in the context of the gender specific upper limits of normality in children (22 U/L for girls and 26 U/L for boys) and not upon the upper limits of normality provided by individual laboratories (14). Reference curves for leptin and adiponectin in the IDEFICS population may be useful when trying to assess the limits of their normality (21).

#### Study Population

All the children in this study were Spanish. Both, children with obesity and children with normal weight were selected from the same schools, and had similar lifestyles. We informed in four schools in the area about this study in order to recruit the participants (Córdoba, Spain). All the parents of the children included in this study gave their written consent, and the study was authorized by the local hospital ethics committee.

First, parents were invited to a briefing on this study and asked to participate in it. All parents with children between the ages of 6 and 9 were summoned. All students whose parents signed the informed consent were included in the study consecutively, by order of registration.

One group included 54 children with obesity [with a body mass index (BMI) exceeding the 95th percentile in the reference tables for the Spanish population] (22), and the control group included an equal number of children with a normal weight (under the 85th percentile) matched by age and sex (aged 6–9 years). Taking ALT as the main variable, and given that the standard deviations for the two groups were 0.061 and 0.083, respectively, and, the expected mean difference was 0.040, to achieve a power of 80% at a confidence level of 95%, we calculated that 54 patients would be required in each of the two groups.

### Anthropometric Measurements and Blood Sampling and Analysis

After a fasting for 12 h, blood samples were collected without venous occlusion from a vein in the antecubital fossa. All the samples were collected between 8:00 a.m. and 9:00 a.m., and were divided into aliquots and immediately frozen at −45°C until their analysis. Serum ALT, aspartate aminotransferase (AST), serum cholinesterase, leptin, adiponectin, soluble intercellular adhesion molecule-1 (sICAM), C-reactive protein (CRP), and interleukin-6 (IL-6) levels, as well as a range of MetS-related variables (glucose, insulin, lipids, and blood pressure) were measured in all the children.

Serum glucose, ALT, AST, BChE, creatine kinase (CK), total cholesterol, and triglycerides (TG) concentrations were measured using a random-access analyser (ADVIA 1800, Siemens Healthcare Diagnostics) with reagents from the same manufacturer. Insulin was quantified using an Access 2-Immunoassay System (Beckman Coulter, Brea, CA, USA). High-density lipoprotein cholesterol (HDL-c) was measured after precipitation of chylomicrons, very low-density lipoproteins, and low-density lipoprotein cholesterol (LDL-c) with phosphotungstic acid and magnesium ions. The LDL-c concentration was calculated using the Friedewald formula.

IR was assessed using the homeostasis model formula for IR (HOMA-IR) based on fasting glucose and insulin concentrations: resistance = [insulin (mU/L) × glucose (mmol/L)]/22.5. Apolipoprotein A1 (Apo A1), apolipoprotein B (Apo B), and CRP were measured by nephelometry (N Antisera to Human Apo AI, Apo B, and N high-sensitivity CRP reagent; Behringwerke AG, Marburg, Germany) in a Dade Behring Analyzer II Nephelometer (Dade Behring, Inc., Deer field, IL, USA).

Antigenic immunoassay methods were used to quantify adiponectin (Quantikine human adiponectin, R&D Systems, Wiesbaden-Norderstedt, Germany), sICAM-1 (IBL Immuno-Biological Laboratories, Hamburg, Germany), leptin (Quantikine human leptin, R&D Systems, Wiesbaden-Norderstedt, Germany), and IL-6 (Quantikine Human IL-6; RD Systems, Wiesbaden-Norderstedt, Germany) in a microtiter plate analyser (Personal LAB, Phadia Spain SL, Barcelona, Spain).

Free fatty acids (FFAs) were quantified by a colorimetric enzyme assay (NEFA C ACS-ACOD Method, Woko Chemicals GmbH, Neuss, Germany). Blood pressure was measured with a mercury sphygmomanometer (Pymah Corporation, Sommerville, NJ, USA) after resting for 20 min in a supine position. The measurements were performed on three consecutive days and the mean was used in our analyses. The sphygmomanometer cuff width had to cover 2/3 of the length of the child's arm and so three cuff sizes were available for use (9 cm × 32 cm, 11 cm × 36 cm, and 12 cm × 41 cm). Weight was measured to the nearest 0.1 kg and height to the nearest 0.1 cm. BMI was calculated as the weight (kg)/height (m)^2^.

### Statistical Analysis

Statistical assessment was performed using Microstat (Ecosoft, Indianapolis, IN, USA) or GraphPad InStat software (GraphPad Software, San Diego, CA, USA). Abnormal values (outliers) were excluded by applying Reed's method. The distribution of each variable was tested to check for deviance from the Gaussian distribution, and the equality of the variance was checked by using Snedecor's *F*-test. The mean values of the groups were compared by applying Student's unpaired *t*-tests. All the results were expressed as a mean ± standard error mean (*SEM*) with a 95% confidence interval (95% CI). Statistical significance was set at *p* < 0.05. The correlation between the variables was evaluated using Pearson's correlation coefficient and regression analysis. Multivariate regression analysis was performed using the stepwise method. For each variable, potential confounders (0.05 < *p* < 0.2) were evaluated by analyzing the raw and adjusted regression coefficients.

## Results

### Liver Enzymes, Insulin Resistance, and Parameters Related to MetS

The clinical, anthropometric, and biochemical parameters related to MetS were measured in both groups (Table 1). The mean ALT levels were significantly higher in children with obesity, at 19.57 U/L (95% CI [18.21–20.93]) compared to 17.20 U/L in the control group (95% CI [16.21–18.19]; Table 2). Although the BChE levels were higher in the children with obesity group at 11,178.5 U/L (95% CI [10,888.9–11,468.2]) compared to 10,636.3 U/L in the control group (95% CI [10,201.1–11,072.1]; Table 2). No significant differences were found in the AST values.

**Table 1:** Comparison between children with obesity and children with normal weight.

	**Children with obesity (*n* = 54)**	**Children with normal weight (*n* = 54)**	***p***
Age (years)	7.95 ± 0.14	7.89 ± 0.12	0.7414
Male/Female	25/29	25/29	
BMI (Kg/m^2^)	23.34 ± 0.28	16.76 ± 0.19	<0.0001
BMI *z*-score	3.30 ± 0.15	0.06 ± 0.01	<0.0001
Waist circumference (cm)	73.39 ± 0.89	57.74 ± 1.16	<0.0001
SBP (mm Hg)	100.99 ± 1.49	89.36 ± 1.23	<0.0001
DBP (mm Hg)	62.34 ± 1.12	52.36 ± 1.07	<0.0001
Total cholesterol (mmol/L)	4.49 ± 0.08	4.41 ± 0.08	0.5006
Triglycerides (mmol/L)	0.76± 0.04	0.59 ± 0.16	0.0002
HDL-cholesterol (mmol/L)	1.32 ± 0.03	1.47 ± 0.04	0.0013
Apolipoprotein A1 (μmol/L)	53.5 ± 0.71	57.86 ± 1.07	0.0001
Apolipoprotein B (μmol/L)	26.43 ± 0.71	23.93 ± 0.36	0.0038
Free fatty acids (mmol/L)	0.548 ± 0.03	0.502 ± 0.02	<0.0001
Glucose (mmol/L)	5.05 ± 0.06	4.78 ± 0.04	0.0003
Insulin (pmol/L)	47.5 ± 2.64	38.68 ± 1.80	0.0066
HOMA-IR	1.554 ± 0.095	1.187 ± 0.059	0.0013

*Clinical and anthropometric measurements, and biochemical parameters related to metabolic syndrome*.

*BMI, body mass index; SBP, systolic blood pressure; DBP, diastolic blood pressure; HOMA-IR, homeostasis model assessment for insulin resistance. Results are expressed as the mean ± S.E.M*.

**Table 2:** Comparison between children with obesity and children with normal weight.

	**Children with obesity (n = 54)**	**Children with normal weight (n = 54)**	***p***
ALT (U/L)	19.57 ± 0.68	17.20 ± 0.49	0.0058
AST (U/L)	24.81 ± 0.43	24.51± 0.53	0.655
BChE (U/L)	11178.5 ± 144.3	10636.3± 217.6	0.0413
IL-6 (pg/mL)	1.71 ± 0.14	1.43 ± 0.27	0.2526
CRP (mg/L)	2.34 ± 0.25	0.89 ± 0.22	<0.0001
sICAM-1 (ng/mL)	284.9 ± 8.84	250.3 ± 6.33	0.0006
Leptin (ng/mL)	19.39 ± 1.37	4.16 ± 0.54	<0.0001
Adiponectin (ng/mL)	9.74 ± 0.68	11.59 ± 0.67	0.0161
Creatine kinase (U/L)	113.39 ± 4.50	117.18 ± 5.98	0.6142

*Hepatic enzymes, adipokines, inflammation, and endothelial biomarkers*.

*ALT, alanine aminotransferase; AST, aspartate aminotransferase; BChE, butyryl cholinesterase; IL-6, interleukin-6; CRP, C-reactive protein; sIACM-1, soluble intercellular adhesion molecule-1. Results are expressed as the mean ± S.E.M*.

**Figure 1:**
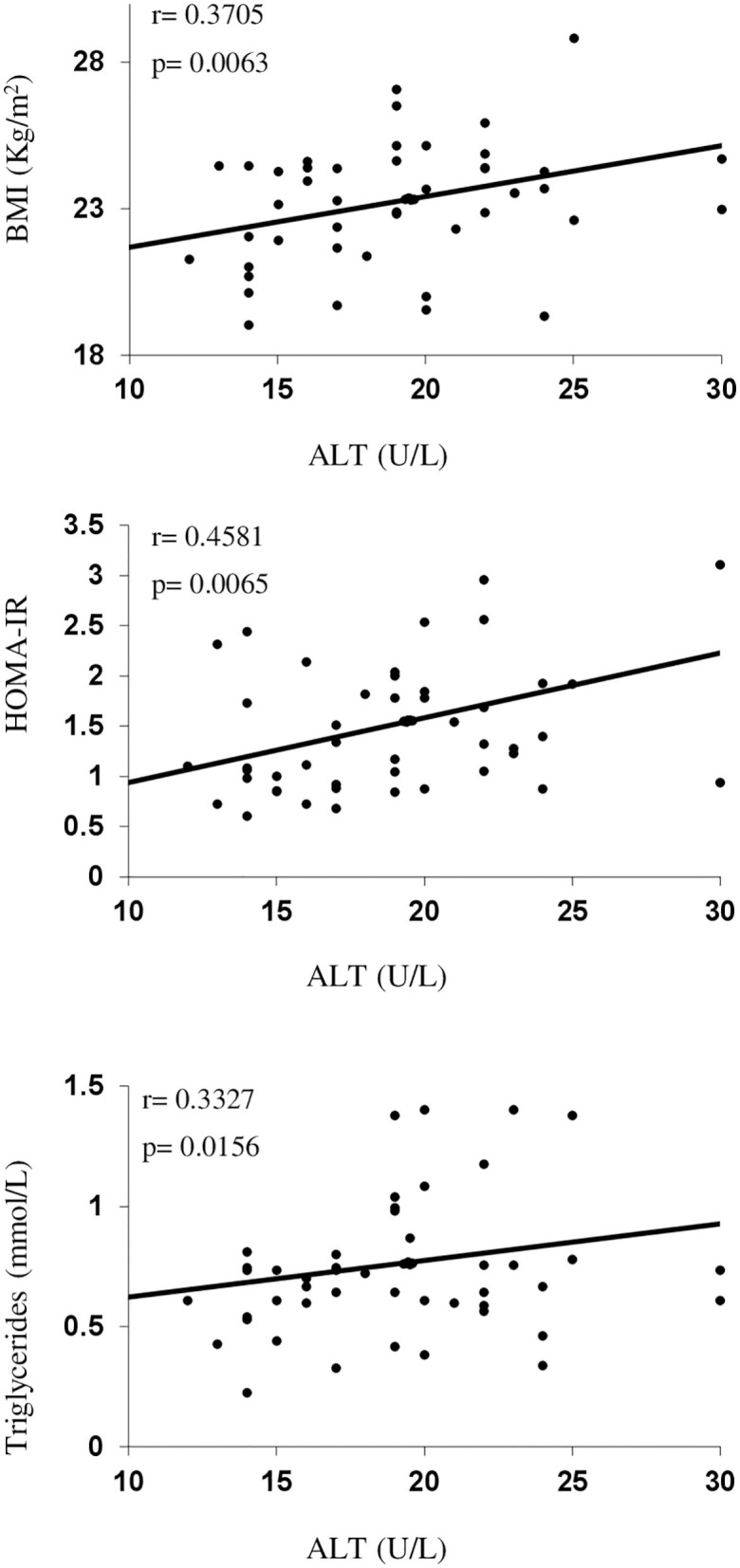
Serum alanine aminotransferase (ALT) levels as a function of body mass index (BMI), homeostasis model assessment for insulin resistance (HOMA-IR), and triglycerides in children with obesity group.

The univariate correlation analysis for MetS-related parameters for the children with obesity group is summarized in Table 3 and Figure 1. In the single linear correlation, ALT and BChE levels correlated with anthropometric measurements, IR, and lipid parameters related to MetS, while the ALT/AST indices correlated only with the anthropometric measurements.

**Table 3:** Single correlation coefficients (*r*) between different variables in the children with obesity group.

	**ALT**		**AST**		**ALT/AST**		**BChE**	
	***r***	***p***	***r***	***p***	***r***	***p***	***r***	***p***
BMI	0.3705	0.0063	−0.1319	0.3466	0.4286	0.0013	0.4733	0.0003
BMI *z*-score	0.3112	0.0247	−0.0616	0.6578	0.2752	0.044	0.3409	0.0124
Waist circumference	0.4057	0.0035	−0.2489	0.0773	0.4057	0.0035	0.3019	0.0257
Glucose	0.3007	0.0286	−0.0597	0.668	0.3128	0.0224	0.0765	0.5822
Insulin	0.4452	0.0007	−0.1293	0.3512	0.4583	0.0005	0.2816	0.0414
HOMA-IR	0.4581	0.0005	−0.1220	0.3795	0.4696	0.0003	0.2739	0.0471
SBP	−0.2270	0.1098	−0.1848	0.1937	−0.0864	0.5463	0.0837	0.5592
DBP	−0.1469	0.3098	−0.1814	0.2012	−0.1090	0.4461	0.1051	0.4629
Total cholesterol	0.07	0.6148	−0.0639	0.6458	0.1116	0.4219	0.0659	0.6391
Triglycerides	0.3327	0.0156	0.0304	0.8318	0.2404	0.0887	0.4199	0.0017
HDL cholesterol	−0.2998	0.0287	−0.0474	0.7394	−0.1990	0.1574	−0.3024	0.0279
Apo A1	−0.3125	0.0237	−0.0057	0.9694	−0.2416	0.0882	−0.2993	0.0289
Apo B	0.1792	0.1947	0.0222	0.8733	0.1708	0.2168	0.0276	0.8429
IL-6	0.3631	0.007	0.0159	0.669	0.2853	0.0365	0.3075	0.0251
CRP	0.4159	0.0018	0.1965	0.1544	0.2795	0.0407	0.3117	0.0243
sICAM-1	0.323	0.0187	0.1332	0.3468	0.3262	0.0181	0.5333	<0.0001
Leptin	0.3633	0.0069	−0.3562	0.0082	0.5336	<0.0001	0.2522	0.0701
Adiponectin	0.0779	0.6058	−0.1961	0.1876	0.1916	0.1587	−0.4327	0.0006

*BMI, body mass index; HOMA-IR, homeostasis model assessment for insulin resistance; SBP, systolic blood pressure; DBP, diastolic blood pressure; Apo A1, Apolipoprotein A1; Apo B, Apolipoprotein B; IL-6, interleukin-6; CRP, C-reactive protein; sIACM-1, soluble intercellular adhesion molecule-1*.

In the children with obesity group, age, and sex-corrected multivariate regression analysis showed that the BMI (*P* partial = 0.0074), waist circumference (*P* partial = 0.0057), serum insulin (*P* partial = 0.0014), HOMA-IR (*P* partial = 0.0009), TG (*P* partial = 0.0287), HDL-c (*P* partial = 0.0369), and Apo A1 (*P* partial = 0.032) were independent predictive factors for ALT. For serum BChE, the age and sex-corrected BMI (*P* partial = 0.0005), waist circumference (*P* partial = 0.0251), TG (*P* partial = 0.0045), and Apo A1 (*P* partial = 0.0327), but not insulin (*P* partial = 0.1071), HOMA-IR (*P* partial = 0.1138), or HDL-c (*P* partial = 0.0599) were independent predictive factors.

In the combined group (children with obesity group and children with normal weight together), serum ALT positively correlated with BMI (*p* = 0.0007), waist circumference (*p* = 0.0009), insulin (*p* < 0.0001), HOMA-IR (*p* < 0.0001), and TG (*p* = 0.0010), and negatively correlated with HDL-c (*p* = 0.0121), and Apo A1 (*p* = 0.0126) while serum BChE positively correlated with BMI (*p* = 0.0007), waist circumference (*p* = 0.0143), insulin (*p* = 0.0494), HOMA-IR (*p* = 0.0320), and TG (*p* = 0.0015), and negatively with HDL-c (*p* = 0.0416).

### Liver Enzymes, Inflammation, Endothelial Dysfunction, and Adipokines

Table 2 shows liver enzymes, adipokines, inflammation, and endothelial biomarkers for children with obesity and children with normal weight. The mean values for CRP, sICAM, and leptin were significantly higher in the children with obesity and adiponectin levels were significantly lower.

**Figure 2:**
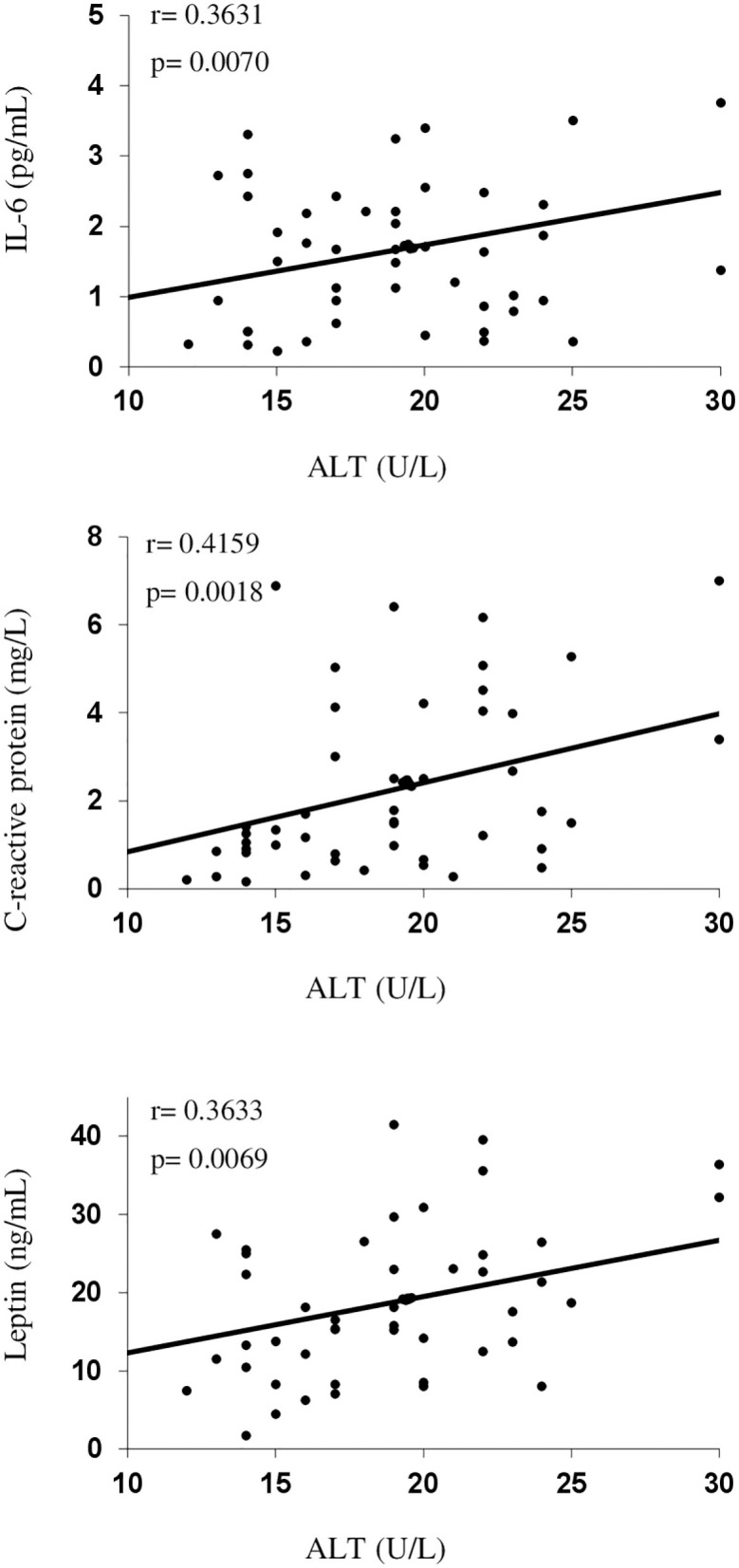
Serum alanine aminotransferase (ALT) levels as a function of interleukin-6 (IL-6), C-reactive protein, and leptin in children with obesity.

The univariate correlation analysis for liver enzymes, adipokines, inflammation, and endothelial biomarkers for children with obesity is summarized in Table 3 and Figure 2. The ALT levels and the ALT/AST index positively correlated with IL-6, CRP, sICAM-1, and leptin, but not with adiponectin values. Serum BChE levels positively correlated with inflammation and endothelial biomarkers and negatively correlated with the adiponectin concentration.

A multivariate regression analysis was carried out for the children with obesity group. When adjusted for age and sex, levels of IL-6 (*P* partial = 0.0090), CRP (*P* partial = 0.0032), sICAM-1 (*P* partial = 0.0224), and leptin (*P* partial = 0.0223) were independent predictive factors for ALT. For serum BChE, age and sex-corrected levels of IL-6 (*P* partial = 0.0481), CRP (*P* partial = 0.0497), sICAM-1 (*P* partial < 0.0001), and Apo A1 (*P* partial = 0.0223) were independent predictive factors.

In the combined children with obesity and children with normal weight group, serum ALT positively correlated with levels of IL-6 (*p* = 0.0040), CRP (*p* = 0.0003), sICAM-1 (*p* = 0.0018), and leptin (*p* < 0.0001), but not with adiponectin (p = 0.5397) while BChE levels positively correlated with IL-6 (*p* = 0.0481), sICAM-1 (*P* partial = 0.0004), and leptin (*p* = 0.0047) levels, and negatively correlated with adiponectin (*p* = 0.0082).

## Discussion

MetS is associated with an increased risk of type 2 diabetes, cardiovascular disease, and all-cause mortality (23). Moreover, children with metabolic syndrome are at an increased risk of cardiovascular disease in adulthood (24). We and other authors have detected low-grade systemic inflammation, alterations indicative of endothelial dysfunction, and altered adipokine levels alongside the disorders that define MetS (4–6) as well as an increase in leptin and decrease in adiponectin in children with obesity. In this work we described an increase in liver-function enzymes in prepubescent children with obesity compared to children with normal weight of an equal age. In addition to the variables related to MetS, we also correlated the values of these enzymes with markers of inflammation, endothelial dysfunction, and adipokine levels.

### Liver Enzymes, Insulin Resistance, and Parameters Related to MetS

Non-alcoholic fatty liver disease (NAFLD) is considered to be the hepatic component of metabolic syndrome (25). In concert with the increase in prevalence rates of obesity and metabolic syndrome, the prevalence of NAFLD has increased dramatically to over 25% of the population worldwide (26).

The prevalence of NAFLD in children overweight or obesity ranges from 29.8 to 34.7% (27) and can cause a broad spectrum of lesions (9). The risk of steatosis and non-alcoholic steatohepatitis increases in children with obesity with MetS (9). Individuals with NAFLD had a significantly higher risk of cardiovascular disease (28).

The high prevalence of NAFLD, and its possible serious health consequences, make its early detection important because simple steatosis is reversible by lifestyle modifications, especially weight loss (29). Some expert committees recommend the use of serum ALT levels to screen for NAFLD in children older than 10 years (14). In this work, we analyzed the biochemical variables related to obesity and NAFLD before puberty. Studying these in this age group also allowed us to study how these parameters affect IR by eliminating the interference produced by hormonal changes during puberty.

ALT is closely related to the accumulation of fat in the liver and is considered a sensitive indicator of liver injury. It has been correlated with obesity and several components of MetS, including dyslipidaemia (30). Its elevation is also associated with the incidence of MetS, diabetes mellitus, and cardiovascular disease (14). Children with increased liver transaminases as surrogates of NAFLD show higher prevalences for prediabetes and type 2 diabetes mellitus as compared to those with normal transaminases (31)

AST and gamma glutamyl transferase (GGT) have not yet been independently tested as screening tools for NAFLD in children. In the context of elevated ALT, higher AST and GGT levels are associated with poorer histology (14). However, elevated AST or GGT in the context of normal ALT can also be representative of conditions other than NAFLD. Different studies describe a high prevalence of NAFLD in children and adolescents aged 10–19 years, even using an upper limit of normality for ALT that is lower than the usual standards (32). They propose that the definition of the upper limit of normal for ALT can be adjusted for each gender and ethnicity in the general population, while the laboratory ALT thresholds used for children should be re-examined. Similar to the results described in adults and adolescents, ALT was increases in prepubescent children with obesity in this study compared to children with normal weight. ALT correlated with anthropometric measurements, IR, and lipid parameters related to MetS and provided evidence that metabolic disorders related to obesity and NAFLD begin at a very early age in children with obesity. However, this association persisted when children with obesity and children with normal weight were jointly analyzed, suggesting that the increase in ALT levels entails a progressive increase in the values of parameters related to MetS.

The ALT test outcome is a continuous variable, and even fluctuation in the normal range also indicates a potential risk of metabolic disorders or cardiovascular disease in a given population (33). Of note, the definition of the upper limit of normality for ALT can be adjusted according to gender and ethnicity in the general population. For some authors, using the gender-specific upper limits of normality in children as a reference, the use of two times the gender-specific ALT in overweight and obese children aged ≥ 10 years showed a sensitivity of 88% and a specificity of 26% for the diagnosis of NAFLD (14, 34). Thus, it might be of interest to review the normal limits of ALT, as well as considering the inclusion of liver enzyme assessments, even for children aged under 10 years.

NAFLD is closely related to IR and hyperinsulinemia, which favors an increase in the levels of free fatty acids, TG, and the onset of hepatic steatosis (35). The group of children with obesity studied in this work presented an increase in FFAs, TG, and IR markers and both serum TG and HOMA-IR were correlated with liver enzyme values. The presence of saturated free fatty acids generates reticulocyte stress and hepatocellular lesions (36). Indeed, an increase in plasma levels of BChE has been reported in individuals with abdominal obesity and MetS (15).

Serum BChE levels are associated with components of MetS has also been described in adolescents (37). In our group, BChE values were also correlated with parameters related to MetS, although when corrected for age and sex, insulin, and HOMA-IR were not independent predictive factors. However, this association was also maintained when both groups were analyzed together. The ALT/AST index did not appear to provide more information than derived from ALT and BChE separately. Thus, we can conclude that increased levels of liver enzymes and parameters related to altered MetS are present at an early age in children with obesity and there is a correlation between them.

### Liver Enzymes, Inflammation, Endothelial Dysfunction, and Adipokines

Adipose tissue interacts with the liver and releases a series of adipokines involved in processes such as inflammation, insulin sensitivity, and NAFLD. An increase in BChE has also been observed in NAFLD. Another mechanism for these associations may be inflammation (38).

Leptin increases IR and the production of fatty acids in hepatocytes and promotes inflammatory and fibrogenic pathways in the liver (39), mechanisms that can contribute to the development of NAFLD. Higher leptin levels are associated with NAFLD and the serum leptin concentration correlates with its severity (40).

In this age group (6–9 years), we found a significant association both between ALT and BChE with markers of inflammation, endothelial dysfunction, and IR, in turn resulting in a positive correlation between liver enzymes and leptin concentration. Serum CRP levels are predictive of NAFLD and have been related to the presence and severity of liver fibrosis (41).

The transition from simple steatosis to non-alcoholic steatohepatitis is accompanied by an additional decrease in adiponectin levels (42). It may have a key role in the relationship between adipose tissue, IR, and inflammation. Although ALT was not associated with adiponectin levels, we did find a significant inverse correlation between adiponectin and BChE. Thus, from an early age, adipose tissue may be involved in the onset of the metabolic disorders that accompany obesity and its complications such as MetS and NAFLD.

The optimal age for NAFLD screening and the need for repeat screenings remain undetermined because of the lack of pediatric studies on the incidence and natural history of NAFLD in young patients. The North American Society of Pediatric Gastroenterology, Hepatology, and Nutrition (NASPGHAN) guidelines recommend the use of serum ALT levels to screen for NAFLD in children, starting from the age of 9 years (14).

The data provided in this study describe the early onset of metabolic disorders related to obesity and NAFLD, which may suggest that screening should be initiated at ages under 10 years, particularly in children with obesity. Thus, it may be of interest to review the normal limits of ALT and to evaluate liver enzymes from prepubertal ages, as well as assessing the usefulness of other possible biochemical markers in pediatric patients. In this regard, extensive studies will be necessary in prepubertal children, including the evaluation of whether weight loss positively affects liver enzymes and its impact on other variables that accompany metabolic syndrome and NAFLD.

The design of this current study did not allow us to establish if there were any sex-related differences in our population. Although this was a limitation, even after age and sex-corrected multivariate regression analysis, the main variables we studied for this age group maintained the same correlations. Only prepubertal children were studied in this current research in order to eliminate the influence of the hormonal changes that occur with puberty.

Another limitation of this work was that we did not perform any imaging studies. Nonetheless, we did aim to assess whether any alterations in biochemical parameters related to obesity and NAFLD were present in children with obesity before puberty and to determine any correlations between them.

Despite recent advances in the understanding of pediatric NAFLD, the evolution, and consequences of this condition are still unclear. The information from the future research would help create clinical programs for early diagnosis and intervention before significant vascular disease begins (43). In addition to the determination of liver enzymes, other parameters related to inflammation and adipokines could also add information to its evaluation in children with obesity at an early age (6–9 years).

## Conclusions

We described an increase in liver enzyme values, markers of IR, inflammation, and endothelial dysfunction, together with the variables that define MetS and altered levels of adipocytokines in prepubescent children with obesity compared to age and sex-matched children with normal weight and there was a correlation between these alterations and liver-function enzymes.

## Data Availability Statement

The original contributions presented in the study are included in the article/supplementary material, further inquiries can be directed to the corresponding author/s.

## Ethics Statement

The studies involving human participants were reviewed and approved by the Research and Ethics Commission, North Sanitary Area of Cordoba, Hospital Valle de los Pedroches, C/ Juan del Rey Calero s/n 14400, Pozoblanco, Córdoba, Spain. Written informed consent to participate in this study was provided by the participants' legal guardian/next of kin.

## Author Contributions

MC is responsible for all data collection and samples of all participants at the Hospital Reina Sofía (Córdoba). She wrote the manuscript and approved the final manuscript as presented. RV-M participated in designing the study, data analysis, interpretation, discussion, and collaborated in the writing of the manuscript. MV and RM created the sampling and supervised data collection. They participated in the interpretation and discussion of the data, reviewed, and edited the manuscript. RC is responsible and coordinator for recruiting children at the Hospital Reina Sofía (Córdoba). LJ-R designed the study, collaborated in data analysis, interpretation, and discussion. All authors have read and approved the final manuscript and assume full responsibility for its contents.

## Conflict of Interest

The authors declare that the research was conducted in the absence of any commercial or financial relationships that could be construed as a potential conflict of interest.

## Abbreviations

MetS: metabolic syndrome
NAFLD: non-alcoholic fatty liver disease
IR: insulin resistance
ALT: alanine aminotransferase
BChE: butyryl cholinesterase
BMI: body mass index
AST: aspartate aminotransferase
sICAM-1: soluble intercellular adhesion molecule-1
CRP: C-reactive protein
IL-6: Interleukin- 6
TG: triglycerides
HDL-c: high-density lipoprotein cholesterol
LDL-c: low-density lipoprotein cholesterol
HOMA-IR: homeostasis model formula for IR
Apo A1: apolipoprotein A1
Apo B: apolipoprotein B
FFAs: free fatty acids
SEM: standard error mean.
